# Buckled MEMS Beams for Energy Harvesting from Low Frequency Vibrations

**DOI:** 10.34133/2019/1087946

**Published:** 2019-08-08

**Authors:** Ruize Xu, Haluk Akay, Sang-Gook Kim

**Affiliations:** Massachusetts Institute of Technology, Mechanical Engineering Department, Cambridge, MA 02139, USA

## Abstract

Vibration energy harvesters based on the resonance of the beam structure work effectively only when the operating frequency window of the beam resonance matches with the available vibration source. None of the resonating MEMS structures can operate with low frequency, low amplitude, and unpredictable ambient vibrations since the resonant frequency goes up very high as the structure gets smaller. Bistable buckled beam energy harvester is therefore developed for lowering the operating frequency window below 100Hz for the first time at the MEMS scale. This design does not rely on the resonance of the MEMS structure but operates with the large snapping motion of the beam at very low frequencies when input energy overcomes an energy threshold. A fully functional piezoelectric MEMS energy harvester is designed, monolithically fabricated, and tested. An electromechanical lumped parameter model is developed to analyze the nonlinear dynamics and to guide the design of the nonlinear oscillator based energy harvester. Multilayer beam structure with residual stress induced buckling is achieved through the progressive residual stress control of the deposition processes along the fabrication steps. Surface profile of the released device shows bistable buckling of 200*μm* which matches well with the amount of buckling designed. Dynamic testing demonstrates the energy harvester operates with 50% bandwidth under 70Hz at 0.5g input, operating conditions that have not been demonstrated by MEMS vibration energy harvesters before.

## 1. Introduction

Energy harvesting from the ambient environment is an attractive power source for the Internet of Things (IoT). Vibration, among other energy sources, such as solar, thermal, chemical, wind, and flow, and human motions, is ubiquitous and can be found in civil structures, machines, and human motions and can provide small energy while other forms of energy are not available. Even though there have been tremendous advances in vibrational energy harvesting, challenges still remain with the state of the art, which is evident with the fact the there are no commercially applicable and successful MEMS energy harvesting devices.

Developing useful and commercially viable energy harvesters should include the scalable and low cost manufacturing to ensure the low cost of the massive number of sensors which are sometimes disposable. The size of the energy harvester needs to be able to fit into the small sensing unit. With the cost and size compatible to the current sensors and processors, the harvester should effectively operate with the ambient vibration environment, which is characterized with the low frequency (<100Hz), low acceleration amplitude (<1g), and unpredictable and widely variable frequency nature (wide bandwidth).

The linear resonators such as a cantilever structure can absorb energy from ambient vibrations effectively at their resonant frequencies, but their gain-bandwidth trade-off limits the operation in the wide band variable ambient vibrations. Most of all, the small size of the MEMS structures increases the resonant frequency significantly compared to macroscale devices, which makes it nearly impossible to harvest energy below 100Hz. 

In pursuing wide bandwidth vibration energy harvesting, various approaches have been sought, such as employing multiple resonators [1, 2], frequency tuning [3–7], parametric resonance [8, 9], and nonlinear resonance [10–19]. These mechanisms have benefits on some aspects but at the same time face challenges and limitations. For instance, active frequency tuning consumes power, the tuning efficiency is low, and the tuning range is limited [11]; employing multiple cantilever beams increases both the size and the cost, and a more complex electric circuit may be necessary; the higher order resonance needs long transient build-up time in parametric resonators [8]. Nonlinear resonance was introduced into MEMS energy harvesters [20, 21], which achieved an ultrawide bandwidth of much higher than 20%. However, the beam stiffening also increases the operating frequency to above 1KHz. Bistable nonlinear oscillator based energy harvesters have been investigated for widening the bandwidth [11–19]. Bistable magnetoelastic structure was first investigated by Moon and Holmes [13]. Erturk et al. [11, 16] used a similar device to achieve an order of magnitude larger power output over a wide frequency range. Bistable nonlinear oscillator based energy harvesters [12, 22] were also reported by exerting an axial compression and forming a buckled beam configuration.

Low frequency operation (<100Hz) has become a key goal in the MEMS energy harvesting research in the past few years. The main reason is that the ambient vibrations typically have low frequency spectrum while small-scale energy harvesters tend to have much higher resonance frequency due to the size. Designs to lower the operation frequency of MEMS energy harvesters include designing new geometries [23–31], using soft materials [30, 32, 33], and upconversion mechanisms [34–38]. The geometry design and soft material approaches are straightforward for lowering the resonance frequency: New geometries such as zigzag beam [31] or S-shaped beam [24] and softer materials lower the stiffness of the structure. The upconversion mechanism can increase the transduction element's vibrating frequency and hence the increase the power while absorbing the energy from lower frequency ambient vibrations. The upconversion method inherits some shortcomings from its design however. The two sets of resonators introduce complexity in the device fabrication and costly assembly is typically required. One of the resonators needs to resonate at low frequencies, which increases the size of that resonator and hence the whole device size. Moreover, the impact or magnetic force-based coupling between the two resonators is prone to suffer from significant energy loss and leads to device's low efficiency.

Bistable oscillators have been reported at macroscale to widen the bandwidth. Bistable oscillator could be constructed with buckled beams [36], magnets and magnetoelastic structure [16], or preshaped structures [39]. Magnet-based bistable oscillators are typically at mesoscale and built with assembly of different mechanical parts. We noticed that the large amplitude oscillations at very low frequencies of the bistable oscillators could be independent of the device size, which makes it favorable for low frequency vibration energy harvesting at MEMS scale. A MEMS bistable beam would allow large amplitude vibrations at low frequencies, while their monolithic fabrication process enables low cost production and small form factor.

A clamped-clamped multilayer buckled beam is designed to be a bistable oscillator for absorbing energy. A piezolayer is embedded in the multilayer beam to convert mechanical energy into electrical energy. The beams in our design experience strain by both axial stretching/compression and bending. The beams experience opposite sign bending along the structure at small deflection, but axial strain dominates when the large vertical displacement of the proof mass is much bigger than the thickness of the thin beam. Then the beam behaves nonlinearly. Especially in the case of microelectromechanical system (MEMS) structures, the beam is sufficiently thin that the bending-based linear strain can be neglected [21]. Residual stresses in microfabricated thin films are intentionally induced in the multilayer MEMS beam structure to result in desired amount of buckling. An electromechanical lumped model with closed form lumped parameters is built to analyze the nonlinear dynamics and to guide the design of the MEMS beam layers and achieve the right amount of residual stress across the multilayer stack. In fabrication process steps, in situ control of the residual stress is implemented after each deposition step to achieve the precise amount of buckling designed. A beam array is designed to eliminate warpages and corrugations in the transverse direction of the beams. Transverse directional corrugation and warpage are the result of transverse stress due to the biaxial stress for the buckling in the longitudinal direction while both ends of the beam are fixed; the beam array is then coupled by a central proof mass to constrain the rotational mode of the suspended structure during oscillations. The fully functional MEMS energy harvester has been fabricated and tested. The surface profile scan demonstrates successfully implemented bistable buckling with designed amount. Power measurements demonstrate wide bandwidth operation, with low frequency, low amplitude vibrations, and verify the design concepts which are elaborated in the following sections.

## 2. Model

Analytical model provides the guideline for design and optimization of the multilayer buckled beam. Previous theoretical works on the modeling of the bistable oscillators or energy harvesters include the following: [40] investigated a bistable Duffing oscillator with electromechanical coupling, while the simulations are only in the time domain; [18] has formulated a PZT patched cantilever beam harvester with magnetic force induced bistability; [12] modeled a buckled beam bistable energy harvester, while the interest is on the stochastic excitation. Composites based bistable plates were modeled in [41, 42]. Even though our design is based on a bistable oscillator, there are some features of the specific design that need to be modeled: the device is targeting to work at low frequencies with continuous harmonic vibrations; it will be implemented by MEMS fabrication with multilayer structure; the buckling is induced by the residual stress of the microfabricated thin films; the piezoelectric layer converts the mechanical energy into electricity. To capture these facets of the design, we developed a theoretical framework with an electromechanically coupled lumped model, which incorporates the multilayer structure and residual stress. The model is solved analytically by harmonic balance to obtain the frequency response of the energy harvesters, which is of our primary interest.

**Figure 1:**
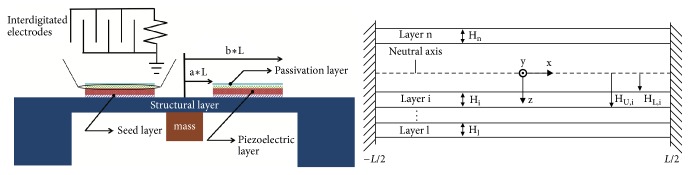
(a) Schematics of the doubly clamped beam based energy harvester. The piezoelectric element is in 33 mode with top interdigitated electrodes. The piezoelectric element and electrode span symmetrically from −*b* · *L* to −*a* · *L* and *a* · *L* to *b* · *L* on the substrate. (b) The beam has *n* layers of thin films in different material with various thicknesses.

The energy harvester we model has a clamped-clamped beam structure of a stack of thin films including structural layer, seed layer, piezoelectric layer, and passivation layer (Figure 1(a)). A heavy proof mass is concentrated at the middle of the beam to capture the external vibration and excites the whole beam to oscillate out of plane. Piezoelectric elements work in 33 mode with top interdigitated electrodes, coupling the electrical response with mechanical deformation. The multilayer beam is designed to buckle by incorporating compressive residual stress in the microfabricated thin films. Statically, the beam is designed to either buckle up or down (two equilibria), and the dynamics become complex when the system is continuously excited in postbuckling regime. To simplify the analysis of the complex problem but still capture the essence of the snapping, the beam's vibration mode is assumed and a one degree-of-freedom model has been constructed. The nonhomogeneous cross-section beam structure has been taken into account by considering different thicknesses and material properties of the layers. Furthermore, residual stress of each layer is built in as part of the stiffness of the beam and induces buckling. The electrical and mechanical domains are coupled with piezoelectric coupling, so that the generated electrical signal can be obtained.

### 2.1. Dynamic Governing Equations

The lumped parameter model is formulated by Lagrange's method. In classical mechanics, the* Lagrangian* is defined as

(1) $$\begin{array}{c} L=KE-PE \end{array}$$

where* KE* is the kinetic energy of the system and* PE* is the potential of the system. In this energy harvester, as in many other vibration energy harvesters, the proof mass is much heavier than the beam's distributed mass, so that the kinetic energy of the system can be approximated as that of the center-concentrated proof mass:

(2) $$\begin{array}{c} KE=\frac{1}{2}m{\dot{w}}^{2} \end{array}$$

where* m* is the proof mass and $\dot{w}$ is the time derivative of the beam center displacement, i.e., the velocity of the proof mass. To find out the thermodynamic potential of the system including the piezoelectric material, we start by considering the electrical enthalpy volume density:

(3) $$\begin{array}{c} {\tilde{H}}_{e}=\frac{1}{2}T_{3}S_{3}-\frac{1}{2}E_{3}D_{3} \end{array}$$

and piezoelectric constitutive equations in *d*_33_ mode [16]

(4) T3=c33ES3−E3e33

(5) D3=e33S3+ε33SE3

where *T*_3_, *S*_3_, *D*_3_, and *E*_3_ are the stress, strain, electric displacement, and electric field in 3-direction respectively; *c*_33_^E^, *e*_33_, and *ε*_33_^*S*^ are the elastic modulus, piezoelectric constant, and permittivity of the piezoelectric material; the superscripts* E* and* S* denote that the parameters are at constant electric field and strain, respectively. Substitute *T*_3_ and *D*_3_ in (3), and add the strain energy contributed by the residual stress *T*_0_, ∫_0_^*S*_3_^*T*_0_*ds* = *T*_0_*S*_3_:

(6) $$\begin{array}{c} {\tilde{H}}_{e}=\frac{1}{2}c_{33}^{E}S_{3}^{2}-e_{33}E_{3}S_{3}-\frac{1}{2}\varepsilon_{33}^{S}E_{3}^{2}+T_{0}S_{3} \end{array}$$

The* Lagrangian* of the system can now be evaluated by integrating the enthalpy density over the beam's volume layer by layer,

(7) $$\begin{array}{c} L=\frac{1}{2}m{\dot{w}}^{2}-\overset{n}{\underset{i=1}{\sum }}{∭}_{v_{i}}{\tilde{H}}_{e,i}dv \end{array}$$

where* v*_*i*_ is the volume of* i*-th layer and* n* is the total number of layers. The strains developed in the beam need to be evaluated before carrying out the integrations in (7). The total strain* S*_*T*_ developed in the beam has two components: bending strain, which changes linearly across the beam thickness, and axial strain due to large deflection,

(8) $$\begin{array}{c} S_{T}=-z\frac{d^{2}\hat{w}}{dx^{2}}+\frac{1}{l}\overset{L/2}{\underset{-L/2}{\int }}\frac{1}{2}{\left(\;\frac{d\hat{w}}{dx}\;\right)}^{2}dx \end{array}$$

where* l* is the beam length. The strain is calculated from the neutral axis of the beam (Figure 1(b)). It should be stressed that the beam composes multilayers with various elastic properties; the neutral axis therefore does not coincide with the midplane. The formula for calculating the position of the neutral axis (distance from the bottom surface) of a general* n*-layer beam is

(9) $$\begin{array}{c} h_{neutral}=\frac{\sum_{i=1}^{n}E_{i}H_{i}\sum_{j=1}^{i}H_{j}-\left(1/2\right)\sum_{i=1}^{n}E_{i}h_{i}^{2}}{\sum_{i=1}^{n}E_{i}h_{i}} \end{array}$$

The beam is designed to vibrate out of plane, and by assuming that it vibrates predominantly in one mode, simplification can be made when evaluating the lumped parameters. The first buckling mode of the beam is adopted, which satisfies the boundary conditions of clamped-clamped beam and has been verified as the vibration mode shape at the largest deflection in experiment [43]. The deflection of the beam can then be separated into time and space,

(10) $$\begin{array}{c} \hat{w}=\frac{w\left(t\right)}{2}\left(\;1+\cos\frac{2\pi x}{l}\;\right) \end{array}$$

where *w*(*t*) is the deflection of the beam center varying with time.* Lagrange* equations are

(11) $$\begin{array}{c} \frac{d}{dt}\left(\;\frac{\partial L}{\partial {\dot{\xi }}_{i}}\;\right)-\frac{\partial L}{\partial \xi_{i}}=Q_{i}^{Force}+Q_{i}^{Dissipation} \end{array}$$

where *ξ*_*i*_ is the* i*-th independent generalized coordinate and *Q*_*i*_^*Force*^ and *Q*_*i*_^*Dissipation*^ are the generalized external force and the generalized dissipative force. We choose the deflection of the midpoint of the beam *w* and the output voltage* V *as the generalized coordinates. The* Lagrange* equation with respect to the first coordinate *w* is then

(12) $$\begin{array}{c} \frac{d}{dt}\left(\;\frac{\partial L}{\partial \dot{w}}\;\right)-\frac{\partial L}{\partial w}=F-b\dot{w} \end{array}$$

Evaluating the integrations in (7) and substituting into (12), the governing equation of the mechanical domain can be obtained and written in a compact form,

(13) $$\begin{array}{c} m\ddot{w}+k_{L}w+k_{N}w^{3}+b\dot{w}+c_{N}wV_{N}+c_{L}V_{L}=F \end{array}$$

where* k*_*L*_,* k*_*N*_,* b*,* c*_*L*_,* c*_*N*_, and* F* are the linear stiffness and nonlinear stiffness of the beam, the mechanical damping coefficient, the linear and nonlinear electromechanical coupling, and the external excitation force, respectively. These lumped parameters are functions of the device dimensions and material properties, which are useful for device design,

(14) kL=2π4W3l3∑i=1nc33,iEHU,i3−HL,i3+π2W2l∑i=1nT0,iHi

(15) kN=π4W8l3∑i=1nc33,iEHi

(16) cL=πe33WHU,P2−HL,P2sin⁡2πb−sin⁡2πalg

(17) cN=π2e33WHpb−alg

where* W*,* H* are the width and thickness,* a* and* b* denote the span of the electrodes on the beam, since they do not cover the whole beam (Figure 1(a)), and *g* is the gap between two electrode fingers; the subscript* p* denotes that the variable is associated with the piezoelectric layer. It should be noted that the linear stiffness has two parts: the first part is from bending of the beam and the second comes from the residual stress. More particularly, when the residual stress is negative (compressive) and large enough, the linear stiffness* k*_*L*_ will be negative, so that (13) becomes a characteristic bistable* Duffing* equation.

The second* Lagrange* equation with respect to the coordinate* V* is

(18) $$\begin{array}{c} \frac{d}{dt}\left(\;\frac{\partial L}{\partial \dot{V}}\;\right)-\frac{\partial L}{\partial V}=\frac{\int Vdt}{R} \end{array}$$

Taking time derivative of the equation gives the governing equation for the electrical domain,

(19) $$\begin{array}{c} C_{0}\left(\;{\dot{V}}_{L}+{\dot{V}}_{N}\;\right)+\frac{V_{L}+V_{N}}{R}=I_{L}+I_{N} \end{array}$$

where $I_{L}=c_{L}\dot{w}$ and $I_{N}=c_{N}w\dot{w}$ are two parts of the electrical current generated by piezoelectric element through coupling and subscripts* L* and* N* denote the linear and nonlinear coupling, respectively; the induced voltages on the electrical port are written in separate parts* V*_*L*_ and* V*_*N*_, due to the fact that they come from two parts of the current, respectively, and have different frequencies due to different coupling, and this differentiation makes the assumptions on their function simple. *C*_0_ is the internal capacitance of the piezoelectric element and is calculated,

(20) $$\begin{array}{c} C_{0}=\frac{W_{eff}L_{eff}H_{p}\varepsilon_{33}^{S}}{2g^{2}} \end{array}$$

where* W*_*eff*_ and* L*_*eff*_ are the effective width and length of the PZT element (area covered by the electrodes) and the number 2 in the denominator is due to the width of one finger electrode and the gaps between electrodes are the same in the designed MEMS device.

**Figure 2:**
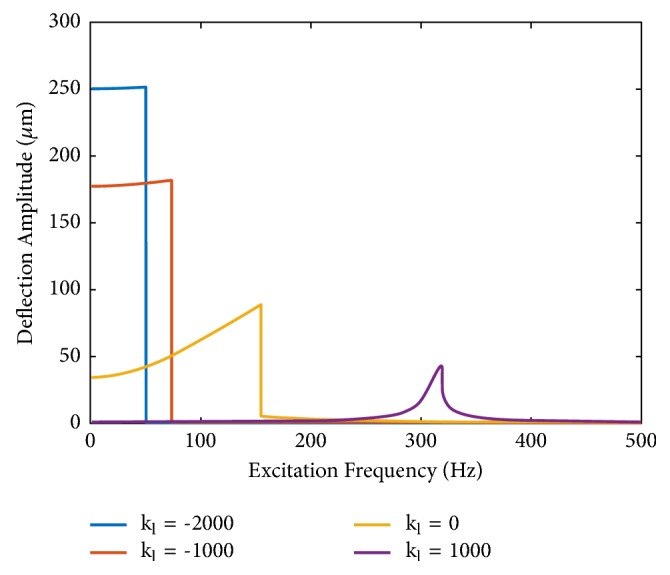
Simulated dynamic responses of the MEMS energy harvester with varying linear stiffness (stress) and other parameters the same. The deflection's amplitude at low frequencies increases with the linear stiffness* k*_*l*_ varying from positive to negative.

The nonlinear governing equations (13) (19) are solved analytically using the harmonic balance method, so that the frequency responses are obtained. Both softening (intrawell) and stiffening (interwell) responses are derived, but the interwell oscillations have larger amplitude and generate more power [43]. The enhancement of the bistable oscillator on the low frequency operation is illustrated in Figure 2, which shows the frequency responses of the MEMS energy harvester with varying linear stiffness and a fixed nonlinear stiffness (this is the case when varying only the residual stress in the beam structure). The frequency response shifts to lower frequency with higher amplitude when the linear stiffness switches from positive to negative. When the negative stiffness's amplitude becomes larger, the response is in ultralow frequency range with significantly larger deflection amplitude, which characterizes the large amplitude snap through bistable nonlinear oscillators. The shift of the frequency response to desirable direction (lower frequency and larger amplitude) by tuning the stiffness provides the critical design knob for the new generation energy harvester.

## 3. Design

### 3.1. Residual Stress Induced Buckling

To adapt to the multilayer structure with residual stress, we consider the load from the stress and the initial buckling shape. The total axial load in the multilayer beam is contributed by the stresses from all the layers,

(21) $$\begin{array}{c} P=\overset{n}{\underset{i=1}{\sum }}T_{0,i}H_{i} \end{array}$$

Therefore, knowing the residual stress of each layer, the thickness could be designed so that the load *P* surpasses the critical buckling load *P*_*c*_ = 4*π*^2^*EI*/*L*^2^ to induce buckling. The requirement for designing the buckling beam is to make the longitudinal compression diminish the bending stiffness at the critical point, so that the linear stiffness becomes zero.

(22) kL2π4W3L3∑i=1nc33,iEHU,i3−HL,i3+π2W2L∑i=1nT0,iHi=0

In the postbuckling regime, the beam buckles in its first mode because higher modes are unstable, with an amplitude *w*_0_ unknown,

(23) $$\begin{array}{c} w\left(\;x\;\right)=\frac{w_{0}}{2}\left(\;1+\cos\frac{2\pi x}{L}\;\right) \end{array}$$

By dropping the time derivatives and the dynamic input in the governing equation of the lumped model, we obtain a static equilibrium equation,

(24) $$\begin{array}{c} k_{L}w_{0}+k_{N}w_{0}^{3}=0 \end{array}$$

The initial buckling amplitude (midpoint of the beam) thus is

(25) $$\begin{array}{c} w_{0}=\sqrt{-\frac{k_{L}}{k_{N}}} \end{array}$$

When the stress distribution of the thin films in the multilayer stack is not symmetric, a moment resulting from the stress distribution is induced as

(26) $$\begin{array}{c} M_{r}=\frac{W}{2}\overset{n}{\underset{i=1}{\sum }}T_{0,i}\left(\;H_{U,i}^{2}-H_{L,i}^{2}\;\right) \end{array}$$

and is minimized in the parametric sweep design process to preserve the bistability.

### 3.2. Threshold of Input Vibration Amplitude

A bistable system's potential energy has double wells with an energy barrier in between [42]. The two wells correspond to the stable equilibria of the system, and the local maximum between the two wells corresponds to an unstable equilibrium. If we consider a buckled beam based bistable system with a linear and a nonlinear stiffness, its potential is

(27) $$\begin{array}{c} {U\left(x\right)}_{Bi-stable}=\frac{1}{2}k_{L}x^{2}+\frac{1}{4}k_{N}x^{4} \end{array}$$

It should be noted that the potential energy of the system is determined solely by the stiffness's, independently of the dynamic state (could be in static state too) or damping. The energy barrier is a function of the linear and nonlinear stiffness:

(28) $$\begin{array}{c} E_{Barrier}=\frac{1}{4}\frac{k_{L}^{2}}{k_{N}} \end{array}$$

The bistable oscillator could oscillate within one potential well, or between the two wells. Since the interwell oscillation gives large amplitude deflection and hence higher power, it is the ideal operational mode for energy harvesting. Determination on whether the system could overcome the energy barrier and to follow the input vibration to have the dynamic snapping becomes critical in the energy harvester design process. A quick conclusion that the input energy should be higher than the energy barrier will not serve as a sufficient design criterion to determine the mode of oscillation since the system's state depends on its initial state as well as the damping after injecting the energy. When the system oscillates within one well already, whether the system overcomes the barrier relies on whether the input vibration provides the extra energy.

The complex dynamics of the bistable system is analyzed by Melnikov's method. The principle of Melnikov's method is to measure the separation between the stable and unstable manifolds in phase space. Papers [44, 45] provide analysis of similar problems, and the analysis here will follow the same fashion. To simplify the analysis, a mechanical bistable system without electromechanical coupling is considered. The governing equation of the bistable system is

(29) $$\begin{array}{c} m\ddot{x}+c\dot{x}+k_{L}x+k_{N}x^{3}=f\left(\;t\;\right) \end{array}$$

where *m* is the mass, *x* is the displacement, *c* is the mechanical damping coefficient, *k*_*L*_ and *k*_*N*_ are linear and nonlinear stiffness, respectively, and *f*(*t*) is the input force as a function of time. The Melnikov function has been derived and a closed form input threshold for the buckled beam oscillator is obtained to aid the optimum design. The derived input acceleration's amplitude threshold is

(30) $$\begin{array}{c} a_{cri}=\frac{k_{L}c}{k_{N}^{1/2}m^{3/2}}\frac{2}{3\pi \omega \mathrm{sech}\left(\pi \omega /2\omega_{0}\right)} \end{array}$$

It should be noted that the first term of the threshold is a function of the linear and nonlinear stiffness, damping, and the proof mass. These can be identified as the key design parameters for designing the low amplitude bistable oscillator based energy harvester. The obtained closed form threshold of input vibration amplitude is a function of frequency so that, combined with the frequency response obtained from the lumped parameter model, the operating frequency and amplitude of the oscillator could be obtained.

### 3.3. Buckled Beam Array

The buckling happens in both longitudinal and transverse directions due to the biaxial compression. The transverse corrugations of the beam structure can diminish the longitudinal buckling and hence the bistability [46]. The strategy to preserve the longitudinal buckling but reduce the transverse buckling is to increase the critical buckling load of the structure in the transverse direction. Even though the compressive stresses in longitudinal and transverse directions are close in amplitude, if it is lower than the critical load in the transverse direction but higher than the critical load in the longitudinal direction, the buckling only happens in the longitudinal direction.

**Figure 3:**
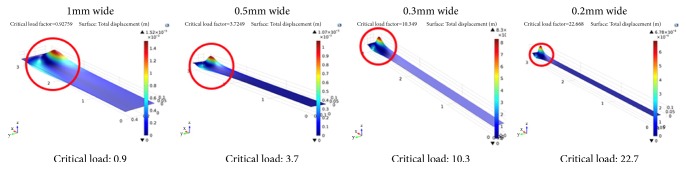
Comsol model of the plate with designed composition and various width. The critical buckling load increases as the plate width decreases.

It could be quickly identified that the width and the length are the critical design parameters for decoupling the buckling in transverse and longitudinal directions, since the beam composition is isotropic across the beam. Analytically, the biaxial buckling could be modeled by the double sinusoidal functions as in the examples in [47], depending on the boundary conditions. The critical loads of a rectangular plate are related to the boundary conditions as well as the width and length: reducing the dimension along the transverse direction increases the critical buckling load in that direction. Therefore, the width of the wide-plate oscillator should be decreased if keeping the same length. The boundary condition combination of the designed structure was more complex than the classical examples in the references and hence the parameter design of the width was aided by finite element analysis with Comsol (Figure 3). Various plate widths of a plate with designed composition are simulated to find the critical buckling load. As can be seen, with deceasing width, the critical buckling load increases (harder to buckle). The plate width of 0.4mm is finally chosen to have the critical buckling load at least 5 times higher than the compressive load to be applied.

The rotation of a narrow beam oscillator about its longitudinal axis is due to the asymmetric distribution of the mass about the beam's longitudinal axis. The relatively large proof mass bonds to the bottom surface of the plate and the center of mass is about half the thickness of the mass away from the plate's longitudinal axis. The vibration of the beam is strictly along the input vibration's direction without rotation only when the center of mass and the plate's longitudinal axis perfectly aligned along the input vibration's direction. If there is a small misalignment, which could be due to the manufacturing or perturbation of the input vibration, the inertial force on the proof mass would produce a torque about the plate's longitudinal axis and trigger the rotation.

With the same input torque *T*, the rotation (*θ*) is inversely proportional to the torsional constant of the plate (*J*_T_) and proportional to the plate's length:

(31) $$\begin{array}{c} \theta =\frac{TL}{GJ_{T}} \end{array}$$

where *G* is the shear modulus of the material and *L* is the length of the plate. For a high aspect ratio of the narrow plate (width/thickness), the torsional constant is *J*_*T*_ = (1/3)*WH*^3^. Since the stack's thickness is determined by the functionality and fabrication compatibility, we could decrease the length of the plate or increase the width of the plate to increase the torsional constant and minimize the rotation. Nevertheless, the length of the plate is also related to the critical buckling load in the longitudinal direction and hence the frequency response, and we chose to increase the width.

**Figure 4:**
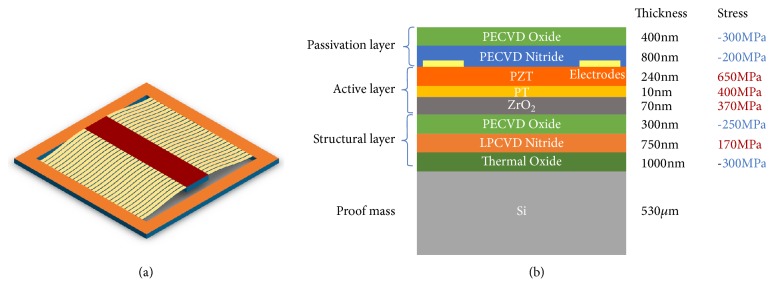
(a) 3D rendition of the buckled beam based energy harvester. The buckling of the beam is exaggerated. (b) Beam composition at the cross-section along the beam center of the MEMS energy harvester.

Summarizing the modifications demanded from the previous analysis, the plate requires small width to increase the critical buckling load in the transverse direction and minimize the buckling in that direction, while the oscillator as a whole should have larger width to resist the rotation. The two seemingly contradictory requirements could be decoupled by coupling multiple single beams to a parallel beam array, so the width of the beam and the width of the beam array are decoupled. The schematic of the new design is illustrated in Figure 4. The narrow beam could minimize the buckling in transverse direction locally, while the much larger width of the beam array including the gap between the single beams increased the torsional constant significantly to restrain the rotations.

## 4. Fabrication

### 4.1. Process Flow

The fabrication process starts with 4" <100> silicon wafers. 300*nm* thermal dioxide is grown with wet oxidation. The thermal dioxide is the bottom layer of the multilayer beam and serves as the etch stop of the final DRIE release. One LPCVD silicon nitride layer (tensile) and one PECVD silicon dioxide layer (compressive) are then deposited. The dual-frequency plasma deposition of the ST Systems CVD enables flexible stress control in a wide range from tensile to compressive. The tuning parameter is the ratio of the duration of the applied high (13.56MHz) and low (380kHz) frequency plasma. The growth rate and residual stress of the PECVD thin films have been characterized by measuring the thickness and the wafer bow before and after the deposition. The data guides the control of the injected compression in the structure and the balance of the stress in the stack. ZrO_2_ and PbTi are the diffusion barrier and the seed layer that were sol-gel spin coated. The PZT solution has a composition of Pb/(Zr+Ti) of 118/100 along with a Zr/Ti ratio of 52/48. This solution is supplied by Mitsubishi Materials as their E1 type PZT sol-gel. The PZT solution is spun on the substrate at 500 rpm for 5s and 3000 rpm for 30 s. The precursor gel film is pyrolyzed at 390°C for 5 min on a hot plate. This deposition process cycle is repeated 2 to 3 times to make a PZT layer of 150 – 240 nm thickness, depending on batch-to-batch process limitations. The PZT film is then annealed at 700°C for 1 min. Electrodes are fabricated by electron beam deposition on a photolithographically patterned interdigitated geometry. 200Å of titanium and 2000Å of gold are deposited, fabricating the electrodes on the PZT layer. PECVD silicon nitride and dioxide as the passivation layers are deposited on top to balance the stress in the stack.

**Figure 5:**
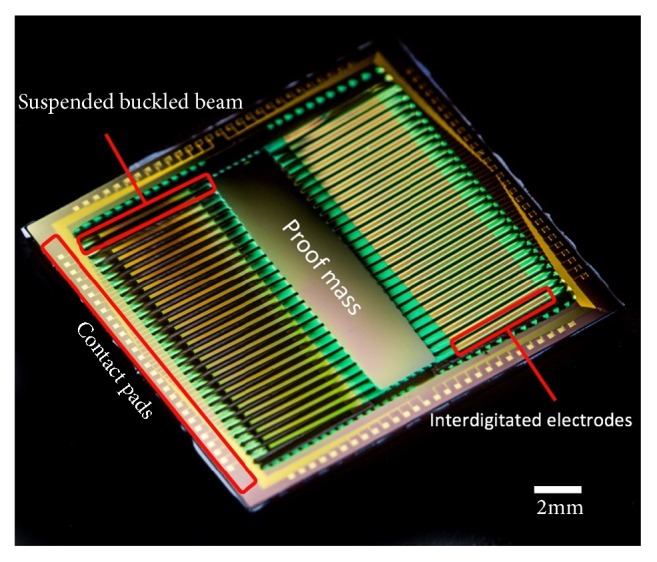
Photo of the released device.

**Figure 6:**
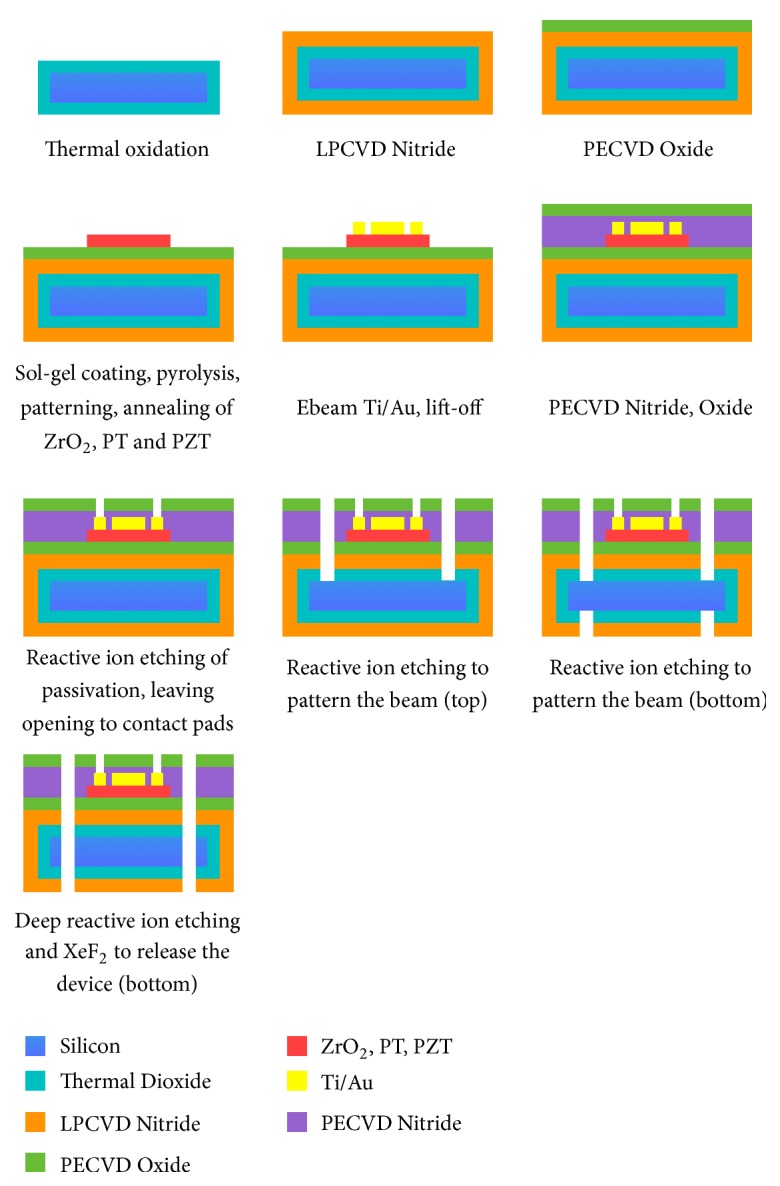
Fabrication process flow of the PZT embedded fully functional energy harvester.

Reactive ion etching (RIE) is used to etch through the whole silicon dioxide and nitride stack from the top to define the beam structure and leave openings to the contact pads and from the backside to pattern the frame and proof mass, leaving opening for the deep reactive ion etching (DRIE). Finally, etching through the whole wafer thickness from the back (DRIE) releases the device. An extra step of XeF_2_ etching is used to gently remove the residual silicon. The released device is shown in Figure 5. The fabrication process flow is shown in Figure 6.

### 4.2. Compression Control of the Multilayer Structure

The residual stress in thin films microfabricated on a substrate causes a change in the radius of curvature of the substrate, which can be measured. The thickness of the dielectric thin films can be measured by Filmetrics. The patterned ZrO2, PT, and PZT layers' thicknesses are measured using surface profilometer. Tencor FLX-2320 at TRL scans the reflected surface of the sample with laser before and after each deposition. The stress can then be calculated from Stoney's formula:

(32) $$\begin{array}{c} \sigma_{f}=\frac{E_{s}H_{s}^{2}}{6RH_{f}\left(1-\nu_{s}\right)} \end{array}$$

where* E*_*sub*_,* υ*_*sub*_, and* h*_*sub*_ are Young's modulus, Poisson's ratio, and thickness of the substrate,* R* is the substrate radius of curvature, and* t*_*film*_ is the thickness of the film. The measured residual stresses of the materials in the MEMS energy harvester device are listed in Table 1.

**Figure 7:**
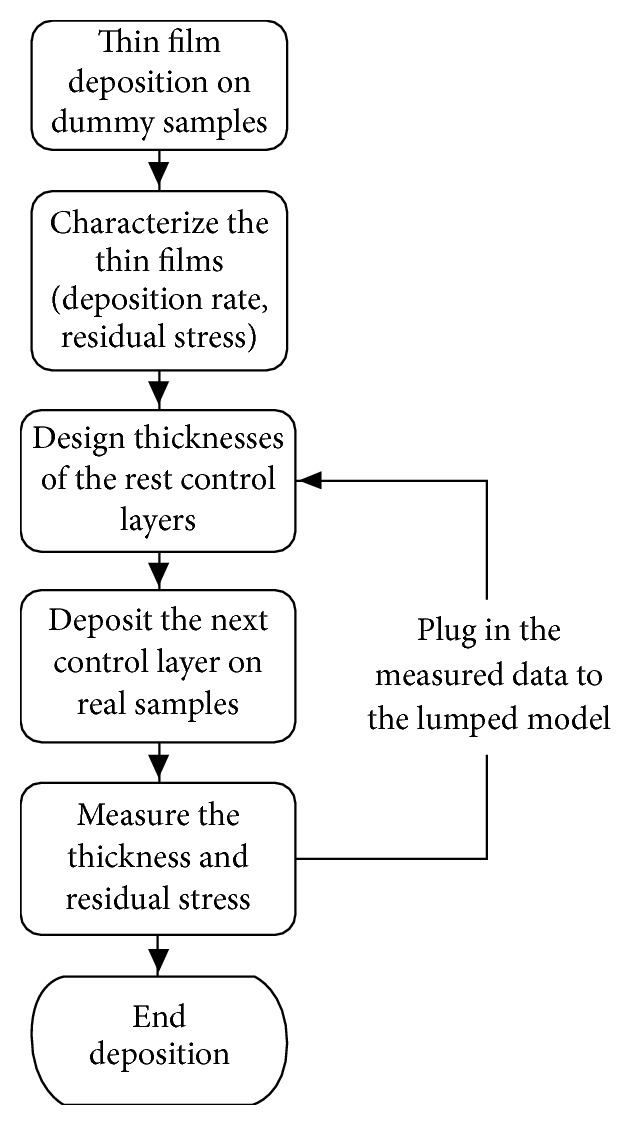
Flow chart of the stress control process with feedback loop.

Even though the deposition rate and the residual stress of each material have been characterized extensively, there is variation in the deposited thin films in different batches. Variation is reduced by a feedback control scheme (as shown in Figure 7). There are three PECVD based control layers: the silicon oxide layer underneath the ZrO2 and the two passivation layers. After the deposition of each layer, the measured thickness and residual stress are fed in the lumped parameter model, and the rest control layers' thickness will be recalculated to adapt to the change in previous deposited layers. In this way, the deviation of the total compression and linear stiffness from the designed value could be minimized.

## 5. Testing

### 5.1. Buckled Geometry Validation

**Figure 8:**
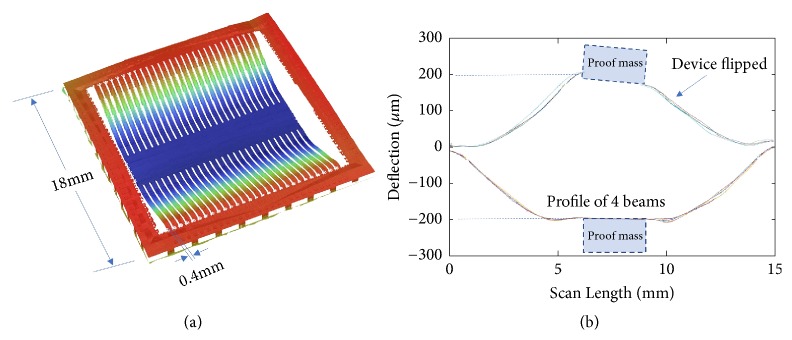
Surface profile scan. (a) Three-dimensional representation of the scanned surface of the whole device. (b) Surface profile of four beams showing the buckling on both sides.

The released devices show buckling in beams that are visible to naked eyes. But to more accurately characterize the buckling and verify the bistability of the buckled structure, optical profiling of the surface is done. Wyko NT9800 optical profilometer is used to scan the surface profile, and the stitching assembles multiple scans to cover the whole beams' surface profile. The surface profile of the whole device is scanned, as shown in Figure 8(a). The 28 beams on the same device show good consistency in buckling, and the measured surface profile matches well with the design (Figure 4). The device's surface is scanned first with top surface facing up and then flipped so the beams buckle to the other direction and the bottom surface of the beams are scanned. Since the weight of the proof mass is equivalent to 1g loading, which is higher than the threshold of the snap, in this way, we can observe the bistable buckling (Figure 8(b)). The buckling in both directions shows similar midpoint deflection within 5% from 200*μ*m, which proves no significant asymmetrical stress distribution that leads to only one direction buckling. The surface profile and the large buckling in longitudinal direction also prove that the narrow beam width effectively eliminates the corrugations in the transverse direction and preserves the buckling in the desired direction.

### 5.2. Dynamic Testing

**Figure 9:**
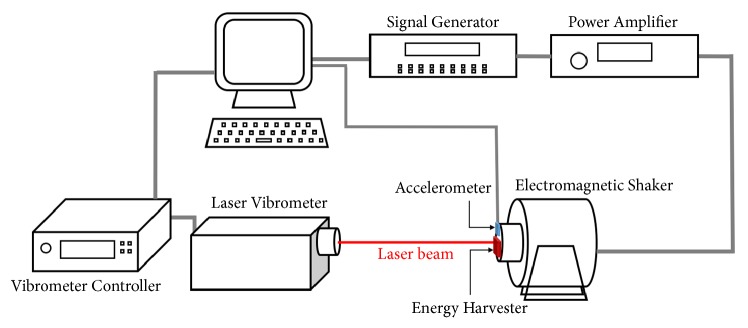
Schematic of the testing setup for measuring the dynamic response of the MEMS prototypes.

The dynamic testing of the device can further validate the design concepts that the buckled beam device could have large amplitude oscillations and with low frequency and low amplitude inputs (<100Hz and 0.5g). The frequency response can be obtained by measuring the deformation of the beam with input vibrations. This testing was done with the setup illustrated by the schematic in Figure 9. The device is mounted on an electromagnetic shaker. The vibration of the shaker is monitored by an accelerometer (Analog Device ADXL335), and the input harmonic signal's frequency and amplitude are controlled by the signal generator (Prema ARB1000) and the power amplifier (Labworks PA138). A laser vibrometer (Polytec Scanning Vibrometer PSV300) was employed to measure the velocity of different spots on the device to calculate the relative movement of the beam to the frame.

**Figure 10:**
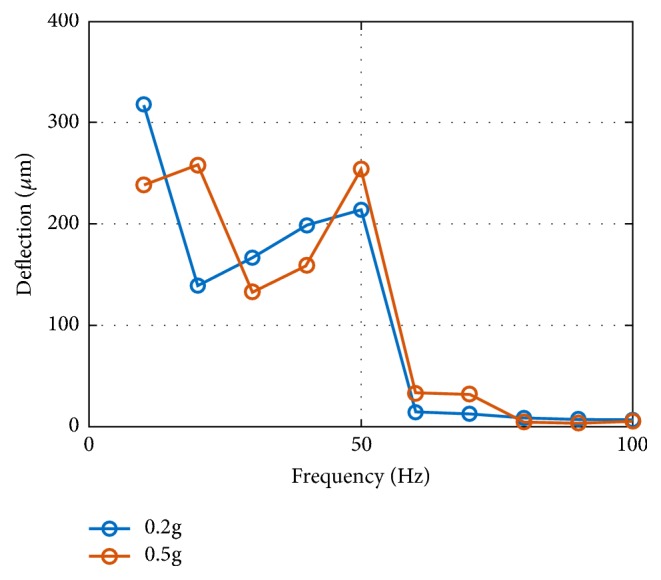
Dynamic testing result: displacement of the mass relative to the frame (silicon mass).

The laser vibrometer measures the velocity of different spots on the energy harvester and calculates the displacement. The input harmonic vibrations are set at fixed frequencies below 100Hz and at different input amplitude level below 0.5g. To eliminate the hysteresis effect, the device is tested with an initial static state, and the input vibration's amplitude increases to reach the target value at a constant frequency. In this way, we can make sure the device starts with lowest energy state and decide if the input energy is enough to trigger the snap, without the interference of the device's initial state. The relative displacement of the mass to the frame is calculated by subtracting the displacement amplitude of the mass from the displacement amplitude of the frame. Since the vibrometer cannot scan the two spots simultaneously but only sequentially, the phase difference cannot be obtained, especially with the abrupt bistable snapping that may not be perfectly the same in each cycle. The estimation is an underestimation as a result, since the phase difference between the two is not always 180°. But the trend shows large amplitude snap in a wide frequency range below 100Hz. With the proof mass of the wafer-thick central silicon proof mass (13.9*mm* × 3*mm* × 525*μm*), the frequency response is shown in Figure 10.

**Figure 11:**
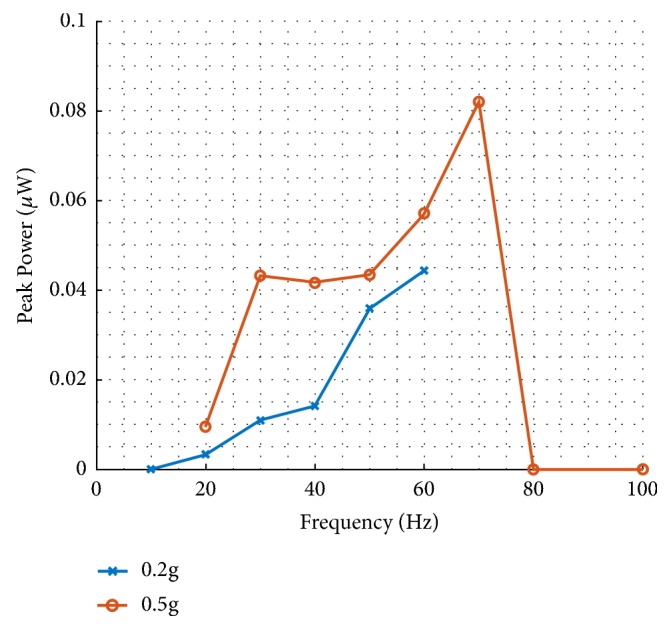
The peak power on a 1MΩ load resistor (with 0.22g tungsten mass).

After postfabrication packaging and prior to dynamic testing, poling of the PZT to align dipoles in the active piezoelectric material was carried out. Poling was conducted for a duration of 30 minutes, at an electric field of 250 kV/cm and an oven temperature of 100°C. The harvester was also connected to a load resistor (1*M*Ω) to measure the power consumed as an indication of the generated power. The voltage across the resistor is measured at a sample rate of 5.2 kHz. The power consumed by the resistor is calculated as *P* = *V*^2^/*R*. During a period of 3.2s, the peak voltage is identified for each fixed frequency and amplitude and is used to calculate the peak power. At 0.5g, the peak power spectrum shows 50% of the jump-down frequency half-power bandwidth from 30Hz to 70Hz (Figure 11).

**Figure 12:**
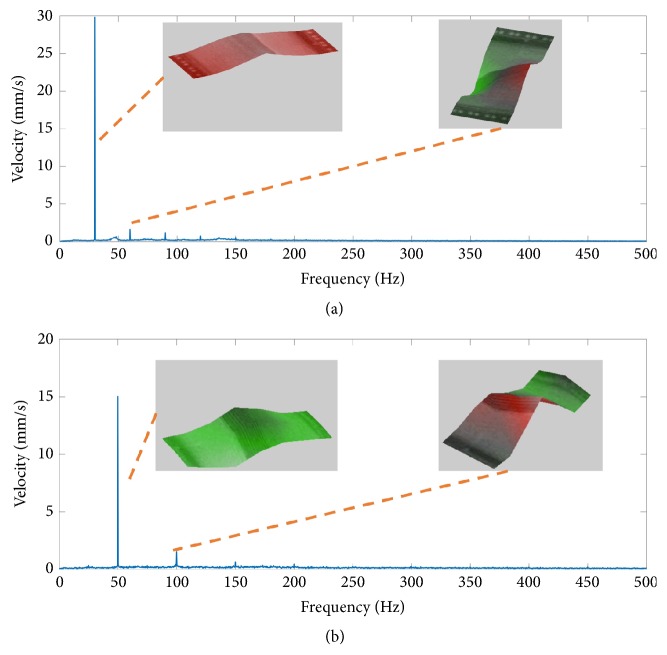
Frequency response of the energy harvester with input amplitude of 0.5g. (a) Excited at 30Hz. (b) Excited at 50Hz.

The device is tested at constant amplitude of 0.5g at 30Hz and 50Hz; as can be seen in Figure 12, the rotation mode has a frequency doubles the drive frequency and has the amplitude less than 1/10th of the primary mode. Figure 12 shows experimentally recorded mode shapes using a laser vibrometer.

### 5.3. Dynamic Optimization

The peak power measured from the device during dynamic testing, plotted in Figure 11, does not meet the power requirements for commercial application involving integration with low-power sensor devices by two orders of magnitude. Given the magnitude of the deflection measured at points along the proof mass, plotted in Figure 10, it follows that these large amplitude oscillations do not efficiently result in axial strain of the piezoelectric beams.

**Figure 13:**
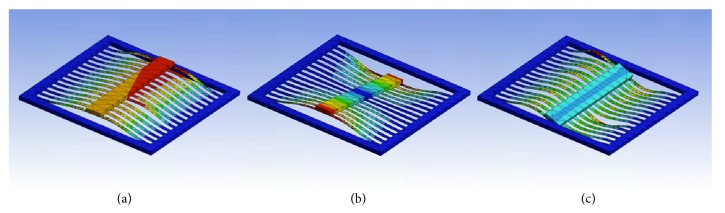
Simulated oscillatory mode shapes. (a) Ideal translational mode. (b) Vertical axis rotational mode. (c) Horizontal axis rotational mode.

Two metrics were used to evaluate the dynamic performance of the buckled beam device. Deflection was measured quantitatively with scanning laser vibrometer focused on an array of locations on the proof mass. The magnitude of proof mass deflection was used to indirectly infer the amount of axial strain experienced by the piezoelectric beams. This deduction assumed that the proof mass's change in position was the result of pure translation in and out of the plane defined by the silicon device frame, illustrated by case(a) in Figure 13. Bending strain (an order of magnitude lower) can potentially cancel out for small oscillations that do not overcome the buckling energy barrier, but these are determined not to induce charge cancellation significantly. Strain from the neutral axis of each beam using the lumped parameter model was estimated assuming all rotational modes of the beams are constrained. Examining the second metric rejected the justification for this assumption. Simulation of the beam dynamic behavior using ANSYS Modal Geometry Analysis package demonstrated the presence of three mode shapes at different resonances. Two of these cases, (b) and (c), suggested the proof mass was unconstrained and had rotational freedom along the two axes defined by the plane of the device frame.

The presence of undesired rotational modes was verified by filming the thin film beams through a microscopic lens and identifying the mode shapes by evaluating the behavior visually in slow motion. The predominant mode of oscillation in addition to the desired translational mode was rotation along the vertical axis, as illustrated in Figure 13 case (b).

A key parameter relating to the magnitude of this mode of rotational freedom is the rotational moment of inertia of the silicon proof mass about the vertical axis. Due to the high aspect ratio of the proof mass's long and narrow geometry, relatively little force is required to induce angular acceleration of the proof mass about this axis. The rotational moment of inertia about the vertical axis for the proof mass is 0.860 g / mm^3^.

A solution to minimize rotational mode of oscillation is to minimize the rotational moment of inertia about the vertical axis. The projected footprint of the proof mass cannot be altered given the requirement to connect to each piezoelectric beam, but its mass can be centralized by reducing the proof mass thickness in all areas with the exception of a central pocket filled with denser tungsten in order to maintain overall mass while reducing rotational inertia. One such iteration of this outlined redesign was demonstrated to reduce the rotational inertia about the vertical axis to 0.485 g / mm^3^. Incorporating the redesigned proof mass into the energy harvester is a key subsequent step to optimizing the dynamic behavior and maximizing power output.

## 6. Conclusion

A bistable nonlinear oscillator based energy harvester is realized and demonstrated at MEMS scale for the first time. The MEMS buckled beam oscillator does not rely on the resonance of the structure but on the snapping motion with large displacement in a wide bandwidth at low frequencies. An electromechanical lumped parameter model of a buckled clamped-clamped multilayer beam with piezoelectric coupling is developed to design and verify the design of MEMS buckled beam harvesters.

A wafer-scale MEMS monolithic fabrication process was successfully completed to make and demonstrate the MEMS buckled beam oscillator for energy harvesting. Residual stress is intentionally introduced and controlled along the MEMS monolithic fabrication processes of 10 thin film layers. With extensive measurements and characterization of the deposition rate and residual stress of each thin film material in the multilayer beam structure, thicknesses of the multiple layers of the beam are designed based on the analytical model developed to incur a desirable amount of compression for buckling of about 200*μm* at the center of the beam. Symmetric distribution of the stress with respect to the neutral axis is also considered to ensure bistable buckling. The thickness and stress of each thin film deposition are monitored during the fabrication as a feedback to adjust the subsequent layer deposition, minimizing the deviation of the final fabricated device from the design. The fabricated device shows buckling matches with the designed amount within 5%. Dynamic testing of the fully functional energy harvester with PZT thin film demonstrates the state-of-the-art operating conditions of MEMS energy harvesters of 50% bandwidth below 70Hz at 0.5g. Further testing with optimized proof mass geometry is expected to boost power output and open the way to commercial application of this MEMS energy harvesting device.

## Figures and Tables

**Table 1:** 

Material	Residual Stress (*MPa*)
Thermal Oxide (300 ~ 1000nm)	−300*MPa* ± 4%
LPCVD Nitride (200nm)	100*MPa*
LPCVD Nitride (700nm)	160*MPa*
LPCVD Nitride (1200nm)	250*MPa*
PECVD Oxide (Low Frequency)	−260*MPa* ± 40%
PECVD Nitride (75% High Frequency)	−240*MPa* ± 40%
ZrO_2_ (90nm)	370*MPa* ± 15%
PT (10nm)	400*MPa* ± 15%
PZT (140 ~ 220nm)	650*MPa* ± 15%

## References

[1] Xue H., Hu Y., Wang Q.-M. (2008). Broadband piezoelectric energy harvesting devices using multiple bimorphs with different operating frequencies. *IEEE Transactions on Ultrasonics, Ferroelectrics and Frequency Control*.

[2] Shahruz S. M. (2006). Design of mechanical band-pass filters for energy scavenging. *Journal of Sound and Vibration*.

[3] Al-Ashtari W., Hunstig M., Hemsel T., Sextro W. (2012). Frequency tuning of piezoelectric energy harvesters by magnetic force. *Smart Materials and Structures*.

[4] Mansour M. O., Arafa M. H., Megahed S. M. (2010). Resonator with magnetically adjustable natural frequency for vibration energy harvesting. *Sensors and Actuators A: Physical*.

[5] Gieras J. F., Oh J.-H., Huzmezan M., Sane H. S. Electromechanical energy harvesting system.

[6] Wu X., Lin J., Kato S. A frequency adjustable vibration energy harvester.

[7] Zhu D., Tudor M. J., Beeby S. P. (2010). Strategies for increasing the operating frequency range of vibration energy harvesters: A review. *Measurement Science and Technology*.

[8] Jia Y., Yan J., Soga K., Seshia A. A. (2013). Multi-frequency operation of a mems vibration energy harvester by accessing five orders of parametric resonance. *Journal of Physics: Conference Series*.

[9] Jia Y., Yan J., Soga K., Seshia A. (2012). *Parametrically Excited Mems Vibration Energy Harvesters*.

[10] Mann B. P., Sims N. D. (2009). Energy harvesting from the nonlinear oscillations of magnetic levitation. *Journal of Sound and Vibration*.

[11] Erturk A., Hoffmann J., Inman D. J. (2009). A piezomagnetoelastic structure for broadband vibration energy harvesting. *Applied Physics Letters*.

[12] Cottone F., Gammaitoni L., Vocca H., Ferrari M., Ferrari V. (2012). Piezoelectric buckled beams for random vibration energy harvesting. *Smart Materials and Structures*.

[13] Holmes P. J., Moon F. C. (1979). A magnetoelastic strange attractor. *Journal of Sound and Vibration*.

[14] Barton D. A. W., Burrow S. G., Clare L. R. (2010). Energy harvesting from vibrations with a nonlinear oscillator. *Journal of Vibration and Acoustics*.

[15] Nguyen D. S., Halvorsen E., Jensen G. U., Vogl A. (2010). Fabrication and characterization of a wideband MEMS energy harvester utilizing nonlinear springs. *Journal of Micromechanics and Microengineering*.

[16] Erturk A., Inman D. J. (2011). Broadband piezoelectric power generation on high-energy orbits of the bistable Duffing oscillator with electromechanical coupling. *Journal of Sound and Vibration*.

[17] Stanton S. C., McGehee C. C., Mann B. P. (2009). Reversible hysteresis for broadband magnetopiezoelastic energy harvesting. *Applied Physics Letters*.

[18] Stanton S. C., McGehee C. C., Mann B. P. (2010). Nonlinear dynamics for broadband energy harvesting: investigation of a bistable piezoelectric inertial generator. *Physica D: Nonlinear Phenomena*.

[19] Hu Y., Xu Y. (2014). A wideband vibration energy harvester based on a folded asymmetric gapped cantilever. *Applied Physics Letters*.

[20] Hajati A. (2010). *Ultra Wide-Bandwidth Micro Energy Harvester [Ph.D. thesis]*.

[21] Hajati A., Kim S.-G. (2011). Ultra-wide bandwidth piezoelectric energy harvesting. *Applied Physics Letters*.

[22] Xu C., Liang Z., Ren B. (2013). Bi-stable energy harvesting based on a simply supported piezoelectric buckled beam. *Journal of Applied Physics*.

[23] Bartsch U., Gaspar J., Paul O. (2010). Low-frequency two-dimensional resonators for vibrational micro energy harvesting. *Journal of Micromechanics and Microengineering*.

[24] Liu H., Lee C., Kobayashi T., Tay C. J., Quan C. (2012). A new S-shaped MEMS PZT cantilever for energy harvesting from low frequency vibrations below 30 Hz. *Microsystem Technologies*.

[25] Liu H., Lee C., Kobayashi T., Tay C. J., Quan C. (2012). A new S-shaped MEMS PZT cantilever for energy harvesting from low frequency vibrations below 30 Hz. *Microsystem Technologies*.

[26] Kim I.-H., Jin S., Jang S.-J., Jung H.-J. (2014). A performance-enhanced energy harvester for low frequency vibration utilizing a corrugated cantilevered beam. *Smart Materials and Structures*.

[27] Xu C., Ren B., Di W. (2012). Cantilever driving low frequency piezoelectric energy harvester using single crystal material 0.71Pb(Mg 1/3Nb 2/3)O 3-0.29PbTiO 3. *Applied Physics Letters*.

[28] Massaro A., De Guido S., Ingrosso I. (2011). Freestanding piezoelectric rings for high e ciency energy har- vesting at low frequency. *Applied Physics Letters*.

[29] Deng J., Rorschach K., Baker E., Sun C., Chen W. (2015). Topology optimization and fabrication of low frequency vibration energy harvesting microdevices. *Smart Materials and Structures*.

[30] Liu W., Han M., Meng B., Sun X., Huang X., Zhang H. (2014). Low frequency wide bandwidth MEMS energy harvester based on spiral-shaped PVDF cantilever. *Science China Technological Sciences*.

[31] Sharpes N., Abdelkefi A., Hajj M. R., Heo J., Cho K.-H., Priya S. (2015). Preloaded freeplay wide-bandwidth low-frequency piezoelectric harvesters. *Applied Physics Letters*.

[32] Edamoto M., Suzuki Y., Kasagi N. (2009). *Low-Resonant-Frequency Micro Electret Generator for Energy Harvesting Application Department of Mechanical Engineering*.

[33] Xie M., Aw K. C., Gao W. (2015). A multi-layered polydimethylsiloxane structure for application in low-excitation, broadband and low frequency energy harvesting. *Sensors and Actuators A: Physical*.

[34] Gu L., Livermore C. (2011). Impact-driven, frequency up-converting coupled vibration energy harvesting device for low frequency operation. *Smart Materials and Structures*.

[35] Burgueno R., Lajnef N. (2014). *Energy Harvesting Devices for Low Frequency Applications*.

[36] Jung S., Yun K. (2010). Energy-harvesting device with mechanical frequency-up conversion mechanism for increased power efficiency and wideband operation. *Applied Physics Letters*.

[37] Pillatsch P., Yeatman E. M., Holmes A. S. (2014). A piezoelectric frequency up-converting energy harvester with rotating proof mass for human body applications. *Sensors and Actuators A: Physical*.

[38] Halim M. A., Park J. Y. (2014). Theoretical modeling and analysis of mechanical impact driven and frequency up-converted piezoelectric energy harvester for low-frequency and wide-bandwidth operation. *Sensors and Actuators A: Physical*.

[39] Qiu J., Lang J., Slocum A. (2004). A curved-beam bistable mechanism. *Journal of Microelectromechanical Systems*.

[40] Erturk A., Inman D. J. (2011). *Piezoelectric Energy Harvesting*.

[41] Arrieta A. F., Delpero T., Bergamini A. E., Ermanni P. (2013). Broadband vibration energy harvesting based on cantilevered piezoelectric bi-stable composites. *Applied Physics Letters*.

[42] Taki M. S., Tikani R., Ziaei-Rad S., Firouzian-Nejad A. (2016). Dynamic responses of cross-ply bi-stable composite laminates with piezoelectric layers. *Archive of Applied Mechanics*.

[43] Xu R., Kim S. (2016). Modeling and experimental validation of bi-stable beam based piezoelectric energy harvester. *Energy Harvesting and Systems*.

[44] Stanton S. C., Mann B. P., Owens B. A. M. (2012). Melnikov theoretic methods for characterizing the dynamics of the bistable piezoelectric inertial generator in complex spectral environments. *Physica D: Nonlinear Phenomena*.

[45] Nayfeh A. H., Mook D. T. (1995). Applied nonlinear dynamics. *Methods*.

[46] Xu R. (2018). *Low-Frequency, Low-Amplitude MEMS Vibration Energy Harvesting, Doctoral Thesis [Doctoral, thesis]*.

[47] Timoshenko S. P., Gere J. M. (1989). *Theory of Elastic Stability*.

